# Malignant tumours in urban Ghana: evidence from the city of Kumasi

**DOI:** 10.1186/s12885-019-5480-0

**Published:** 2019-03-25

**Authors:** Yaw Ampem Amoako, Baffour Awuah, Rita Larsen-Reindorf, Fred Kwame Awittor, Gloria Kyem, Kwame Ofori-Boadu, Ernest Osei-Bonsu, Dennis Odai Laryea

**Affiliations:** 1 Department of Medicine, Komfo Anokye Teaching Hospital, P O Box 1934, Kumasi, Ghana; 2 Kumasi Cancer Registry, Kumasi, Ghana; 3 Directorate of Oncology, Komfo Anokye Teaching Hospital, Kumasi, Ghana; 4 Directorate of Ear, Nose and Throat, Komfo Anokye Teaching Hospital, Kumasi, Ghana; 5 Kumasi South Hospital, Atonsu-Agogo, Kumasi, Ghana; 6 Non-Communicable Disease Control Programme, Ghana Health Service, Accra, Ghana

**Keywords:** Cancer, Incidence, Population based cancer registry, Kumasi, Ghana

## Abstract

**Background:**

Data from population-based cancer registries (PBCRs) are a useful resource for estimating the incidence of cancers. PBCR data is useful in the planning and implementation of cancer prevention and control strategies. Ghana’s plan for control of non-communicable diseases recognises the need for good quality data to facilitate the attainment of set goals.

**Methods:**

We reviewed data from the Kumasi Cancer Registry for the year 2015. Data collected included clinical and demographic information, laboratory reports and source of case information. Data was entered into the Canreg-5 software. Data was initially analysed using Canreg-5 to estimate the incidence and age standardised rates (ASR) for various tumours. Data was also exported to Microsoft Excel for further analysis using Epi Info version 7.1.4. Microsoft Excel was used to generate charts and graphs. Aggregated data for the years 2013 and 2014 were also analysed for trends in cancer incidence and ASR.

**Results:**

A total of 736 cancer cases were recorded among the residents of Kumasi for the year 2015. Females accounted for 62.4% of all cases. The overall incidence of cancer in Kumasi for 2015 was 46.1 per 100,000. The mean age of all cases was 51.3 years (with a range of 1 to 99 years). The incidence among female residents was estimated at 54.1 per 100,000 compared with 37.1 per 100,000 in males. Among females, breast and cervical cancers recorded the highest incidences of 16.1 per 100,000 and 13.7 per 100,000 respectively. Among males, prostate cancer had the highest incidence of 10.5 per 100,000. Breast, cervical and liver cancers were the commonest in both sexes accounting for 19.7, 14.7 and 11.4% of cases respectively.

**Conclusion:**

There has been significant improvement in data quality and coverage since the inception of our PBCR in 2012. PBCRs are feasible; therefore there is the need for more such registries to improve data on cancers in Ghana. Consistent with other evidence, we found breast cancer as the commonest female cancer in Ghana.

## Background

Cancer is a major cause of mortality worldwide. In developed countries, cancers are the second leading cause of death but ranks third in low and middle-income countries [1]. The International Agency for Research on Cancer (IARC) projects that an estimated 21.7 million cancer cases will be recorded globally by 2030 with cancer-related deaths projected to hit 13 million [2]. These projections also estimate that cancers will become major causes of morbidity and mortality in developing countries.

There is progress towards combating infectious diseases particularly those related to cancers such as Hepatitis B virus and the Human Papilloma Virus (HPV); these are responsible for some common cancers in sub-Saharan Africa [3, 4]. The prevalence of cervical HPV infections has been found to be high among some sections of the Ghanaian population [5]. The prevalence of other viral infections such as Human Herpes Virus-8 (HHV8), Cytomegalovirus (CMV) and Epstein Barr Virus (EBV) is also high [6]. High levels of cigarette smoke have been found in public smoking places in Ghana [7] with implications for both patrons and the staff working there.

In all these projections from IARC [2] there is an inherent lack of cohesive data on cancers in Ghana. Current estimates of cancer in Ghana are based on mathematical modelling which includes the prevalence of risk factors and the use of reliable data from other countries in Sub-Saharan Africa. In Ghana, some attempts have been made at providing data on cancers but these have largely been hospital-based [8–13]. Estimating the incidence of cancer requires data from population-based cancer registries (PBCRs) [14]. Such registries will have well-defined geographic area(s) of operation. PBCR data is useful in the planning and implementation of cancer prevention and control strategies. The IARC estimates that 1 in 5 low- and middle-income countries have reliable data sources for cancer prevention and control activities [15]. Despite the importance of PBCRs, it is recognised that developing countries have challenges establishing PBCRs and therefore hospital-based cancer registries (HBCR) can be useful in the absence of PBCRs [16]. Ghana’s plan for control of non-communicable diseases recognises the need for good quality data if any significant achievements are to emerge from this policy [17]. We present data on cancers in the city of Kumasi for the year 2015 from the Kumasi Cancer Registry which is currently the only PBCR in Ghana.

## Methods

We reviewed data from the Kumasi Cancer Registry for the year 2015. The Kumasi Cancer Registry is a PBCR established in 2012 to report cancer cases in the population of Kumasi. The registry covers the city of Kumasi, the capital of the Ashanti Region of Ghana. Kumasi is the second largest city in Ghana and is home to the second largest teaching hospital, the Komfo Anokye Teaching Hospital (KATH). The registry collects data routinely for cancers reported among residents of Kumasi from multiple sources. The sources and methods of collection of data for the registry have been previously published [18]. KATH remains the major source of data for the registry largely because it is the biggest facility in Kumasi and also the only one fully equipped to provide radiotherapy and other specialized oncology services in the metropolis. The other sources of data for the registry are the major public hospitals in Kumasi with the requisite human resource to diagnose cancer. In addition, the registry collects data from pathology laboratories (private and public) and from the Births and Deaths Registry. Data collection involves the use of an adapted standard data abstraction form from the African Cancer Registry Network (AFCRN) [19].

Data collection combines passive and active surveillance methods. Active surveillance is undertaken in KATH and involves daily collection of data from the Oncology, Surgery, Medicine, Obstetrics and Gynaecology, Eye, Ear-Nose and Throat, Child and Oral Health departments. Officers review out- and in-patient record books to identify cases of cancer. The record books are reviewed and folders for patients with a documented diagnosis of cancer are retrieved and the relevant data abstracted. Where diagnosis is presumptive, the case is followed up to the relevant laboratory to retrieve the laboratory report. Passive data collection is scheduled every quarter for the other major health facilities in Kumasi. Data abstraction follows a similar procedure with reviews undertaken for both in- and out-patient records. To ensure completeness, records review at the Births and Deaths Registry is undertaken weekly, with the data collected traced back to various wards, departments and/or facilities to ascertain the definite basis for diagnosis. When no trace can be made, especially in instances where the patient dies on the way to the hospital, the basis of diagnosis is captured as ‘death certificate only’. Pathology reports are reviewed weekly from all relevant laboratories including the KATH pathology laboratory. Data collected covered clinical and demographic information, laboratory/ pathology reports and source of case information. Data is entered into the Canreg-5 software developed by IARC. This software has in-built features which use algorithms of selected variables to detect duplications in which case the already entered data is updated with relevant information. Data entered by registrars are validated by a supervisor for confirmation into the database.

Data on the estimated mid-year population of Kumasi for each year was obtained from the Ghana Statistical Service based on the 2010 National Population and Housing Census and the projected growth rates for the city of Kumasi [20]. The reference population was the World Standard Population (Table 1). The International Classification of Diseases (ICD) for Oncology 3rd edition was used to define cancers [21]. No multiple primaries are included in our data as there were none seen over the period.

**Table 1:** Frequency, age specific incidence rates, average annual crude incidence rates and ASR by site in females in Kumasi, 2015

Site/Age group	All Ages	Age unkwnon	0	5	10	15–19	20–24	25–29	30–34	35–39	40–44	45–49	50–54	55–59	60–64	65–69	70–74	75+	Crude Rate	%	Cum (0–64)	CUM (0–74)	ASR	ICD
Lip	1	0	–	–	–	–	–	–	–	–	–	–	–	–	–	–	–	3.6	0.1	0.2	0.00	0.00	0.1	*C00*
Tongue	0	0	–	–	–	–	–	–	–	–	–	–	–	–	–	–	–	–	0.0	0.0	0.00	0.00	0.0	*C01–02*
Mouth	2	0	–	–	–	–	–	–	–	–	1.7	–	–	3.9	–	–	–	–	0.2	0.4	0.03	0.03	0.3	*C03–06*
Salivary glands	2	0	–	–	–	–	–	–	–	–	–	–	2.4	–	–	6.9	–	–	0.2	0.4	0.01	0.05	0.3	*C07–08*
Tonsil	1	0	–	–	–	–	–	–	–	–	–	2.2	–	–	–	–	–	–	0.1	0.2	0.01	0.01	0.1	*C09*
Other oropharynx	0	0	–	–	–	–	–	–	–	–	–	–	–	–	–	–	–	–	0.0	0.0	0.00	0.00	0.0	*C10*
Nasopharynx	2	0	–	–	0.7	–	–	–	–	–	1.7	–	–	–	–	–	–	–	0.2	0.4	0.01	0.01	0.2	*C11*
Hypopharynx	0	0	–	–	–	–	–	–	–	–	–	–	–	–	–	–	–	–	0.0	0.0	0.00	0.00	0.0	*C12–13*
Pharynx unspecified	0	0	–	–	–	–	–	–	–	–	–	–	–	–	–	–	–	–	0.0	0.0	0.00	0.00	0.0	*C14*
Oesophagus	0	0	–	–	–	–	–	–	–	–	–	–	–	–	–	–	–	–	0.0	0.0	0.00	0.00	0.0	*C15*
Stomach	14	0	–	–	–	–	–	–	–	–	–	4.4	2.4	–	–	27.6	20.7	3.6	1.1	2.8	0.04	0.30	1.8	*C16*
Small intestine	0	0	–	–	–	–	–	–	–	–	–	–	–	–	–	–	–	–	0.0	0.0	0.00	0.00	0.0	*C17*
Colon	8	0	–	–	–	–	–	–	–	1.4	3.4	–	–	–	8.3	13.8	5.2	–	0.7	1.8	0.07	0.16	1.1	*C18*
Rectum	4	0	–	–	–	–	–	–	–	1.4	–	2.2	–	–	4.1	–	–	3.6	0.3	0.9	0.04	0.04	0.5	*C19–20*
Anus	5	0	–	–	–	–	–	–	–	–	–	–	4.7	3.9	–	–	–	7.2	0.4	1.1	0.04	0.04	0.5	*C21*
Liver	28	0	–	0.7	–	–	1.7	2.8	2.4	4.2	3.4	2.2	4.7	11.8	4.1	27.6	15.5	3.6	2.3	6.1	0.19	0.41	3.2	*C22*
Gallbladder etc.	0	0	–	–	–	–	–	–	–	–	–	–	–	–	–	–	–	–	0.0	0.0	0.00	0.00	0.0	*C23–24*
Pancreas	3	0	–	–	–	–	–	–	–	–	–	–	2.4	3.9	4.1	–	–	–	0.2	0.7	0.05	0.05	0.4	*C25*
Nose, sinuses etc.	1	0	–	–	0.7	–	–	–	–	–	–	–	–	–	–	–	–	–	0.1	0.2	0.00	0.00	0.1	*C30–31*
Larynx	1	0	–	–	–	–	–	–	–	–	–	–	–	–	4.1	–	–	–	0.1	0.2	0.02	0.02	0.2	*C32*
Trachea, bronchus and lung	5	0	–	–	–	–	0.9	–	–	–	–	–	–	–	8.3	–	–	7.2	0.4	1.1	0.05	0.05	0.5	*C33–34*
Other thoracic organs	0	0	–	–	–	–	–	–	–	–	–	–	–	–	–	–	–	–	0.0	0.0	0.00	0.00	0.0	*C37–38*
Bone	4	0	–	–	–	–	–	1.9	–	1.4	–	–	–	–	4.1	–	–	–	0.3	0.9	0.04	0.04	0.4	*C40–41*
Melanoma of skin	1	0	–	–	–	–	–	–	–	–	–	–	–	–	–	–	–	3.6	0.1	0.2	0.00	0.00	0.1	*C43*
*Other skin*	*2*	*0*	*–*	*–*	*–*	*–*	*–*	*–*	*–*	*–*	*1.7*	*–*	*–*	*–*	*–*	*6.9*	*–*	*–*	*0.2*	*0.4*	*0.01*	*0.04*	*0.3*	*C44*
Mesothelioma	0	0	–	–	–	–	–	–	–	–	–	–	–	–	–	–	–	–	0.0	0.0	0.00	0.00	0.0	*C45*
Kaposi sarcoma	1	0	–	–	–	–	–	–	–	–	–	–	2.4	–	–	–	–	–	0.1	0.2	0.01	0.01	0.1	*C46*
Connective and soft tissue	6	0	–	–	0.7	1.6	–	–	–	–	–	2.2	–	3.9	4.1	–	–	–	0.5	1.3	0.06	0.06	0.7	*C47,C49*
Breast	138	0	–	–	–	–	2.6	1.9	9.5	14.0	30.4	50.1	56.8	63.1	41.4	34.5	20.7	46.8	11.3	30.0	1.36	1.64	16.1	*C50*
Vulva	3	0	–	–	–	–	–	–	–	–	–	–	–	3.9	4.1	–	–	3.6	0.2	0.7	0.04	0.04	0.4	*C51*
Vagina	0	0	–	–	–	–	–	–	–	–	–	–	–	–	–	–	–	–	0.0	0.0	0.00	0.00	0.0	*C52*
Cervix uteri	107	0	–	–	–	–	–	0.9	2.4	7.0	16.9	30.5	23.7	63.1	58.0	75.9	31.1	4.8	8.9	23.4	1.01	1.55	13.7	*C53*
Corpus uteri	10	0	–	–	–	–	–	–	1.2	–	–	–	–	7.9	8.3	13.8	15.5	–	0.8	2.2	0.09	0.23	1.4	*C54*
Uterus unspecified	4	0	–	–	–	–	–	–	–	–	–	–	7.1	3.9	–	–	–	–	0.3	0.9	0.06	0.06	0.5	*C55*
*Ovary*	*29*	*0*	*–*	*–*	*–*	*–*	*0.9*	*1.9*	*4.7*	*1.4*	*5.1*	*4.4*	*11.8*	*7.9*	*–*	*27.6*	*5.2*	*4.4*	*2.4*	*6.3*	*0.19*	*0.35*	*3.3*	*C56*
Other female genital organs	0	0	–	–	–	–	–	–	–	–	–	–	–	–	–	–	–	–	0.0	0.0	0.00	0.00	0.0	*C57*
Placenta	5	0	–	–	–	–	1.7	0.9	1.2	–	1.7	–	–	–	–	–	–	–	0.4	1.1	0.03	0.03	0.4	*C58*
Kidney	4	0	0.6	–	–	0.8	–	0.9	–	–	–	–	2.4	–	–	–	–	–	0.3	0.9	0.02	0.02	0.3	*C64*
Renal pelvis	0	0	–	–	–	–	–	–	–	–	–	–	–	–	–	–	–	–	0.0	0.0	0.00	0.00	0.0	*C65*
Ureter	0	0	–	–	–	–	–	–	–	–	–	–	–	–	–	–	–	–	0.0	0.0	0.00	0.00	0.0	*C66*
*Bladder*	*11*	*0*	*–*	*–*	*–*	*–*	*–*	*–*	*–*	*–*	*1.7*	*–*	*4.7*	*7.9*	*4.1*	*6.9*	*5.2*	*0.8*	*0.9*	*2.4*	*0.09*	*0.15*	*1.3*	*C67*
Other urinary organs	0	0	–	–	–	–	–	–	–	–	–	–	–	–	–	–	–	–	0.0	0.0	0.00	0.00	0.0	*C68*
Eye	3	0	1.9	–	–	–	–	–	–	–	–	–	–	–	–	–	–	–	0.2	0.7	0.01	0.01	0.2	*C69*
*Brain, nervous system*	*7*	*0*	*–*	*–*	*0.7*	*0.8*	*–*	*0.9*	*–*	*4.2*	*1.7*	*–*	*–*	*–*	*–*	*–*	*–*	*–*	*0.6*	*1.5*	*0.04*	*0.04*	*0.6*	*C70–72*
Thyroid	3	0	–	–	–	–	–	–	–	–	–	–	–	3.9	4.1	–	5.2	–	0.2	0.7	0.04	0.07	0.4	*C73*
Adrenal gland	0	0	–	–	–	–	–	–	–	–	–	–	–	–	–	–	–	–	0.0	0.0	0.00	0.00	0.0	*C74*
Other endocrine	0	0	–	–	–	–	–	–	–	–	–	–	–	–	–	–	–	–	0.0	0.0	0.00	0.00	0.0	*C75*
Hodgkin disease	4	0	–	–	0.7	–	–	0.9	–	–	–	2.2	–	–	–	–	–	3.6	0.3	0.9	0.02	0.02	0.3	*C81*
Non-Hodgkin lymphoma	21	0	0.6	0.7	0.7	1.6	0.9	–	1.2	2.8	6.8	2.2	4.7	–	8.3	–	–	10.8	1.7	4.6	0.15	0.15	2.0	*C82–85,C96*
Immunoproliferative diseases	0	0	–	–	–	–	–	–	–	–	–	–	–	–	–	–	–	–	0.0	0.0	0.00	0.00	0.0	*C88*
Multiple myeloma	3	0	–	–	–	–	–	–	–	–	–	–	–	3.9	–	–	10.4	–	0.2	0.7	0.02	0.07	0.4	*C90*
Lymphoid leukaemia	3	0	0.6	0.7	–	–	–	0.9	–	–	–	–	–	–	–	–	–	–	0.2	0.7	0.01	0.01	0.2	*C91*
Myeloid leukaemia	7	0	1.2	–	0.7	–	–	–	–	–	3.4	–	–	7.9	–	–	–	–	0.6	1.5	0.07	0.07	0.7	*C92–94*
Leukaemia unspecified	0	0	–	–	–	–	–	–	–	–	–	–	–	–	–	–	–	–	0.0	0.0	0.00	0.00	0.0	*C95*
*Myeloproliferative disorders*	*0*	*0*	*–*	*–*	*–*	*–*	*–*	*–*	*–*	*–*	*–*	*–*	*–*	*–*	*–*	*–*	*–*	*–*	*0.0*	*0.0*	*0.00*	*0.00*	*0.0*	*MPD*
*Myelodysplastic syndromes*	*0*	*0*	*–*	*–*	*–*	*–*	*–*	*–*	*–*	*–*	*–*	*–*	*–*	*–*	*–*	*–*	*–*	*–*	*0.0*	*0.0*	*0.00*	*0.00*	*0.0*	*MDS*
Other and unspecified	8	0	–	–	–	0.8	–	1.9	–	–	–	2.2	4.7	–	4.1	–	–	3.6	0.7	1.8	0.07	0.07	0.8	*O&U*
All sites	459	0	5.0	2.0	5.1	5.6	8.5	16.0	22.5	37.9	79.5	104.6	134.9	201.2	174.0	241.6	134.6	90.9	38.0		4.00	5.89	54.1	*ALL*
All sites but C44	457	0	5.0	2.0	5.1	5.6	8.5	16.0	22.5	37.9	77.8	104.6	134.9	201.2	174.0	234.7	134.6	190.9	37.9	100.0	3.99	5.85	53.8	*ALLbC44*

Reference population: world standard PopulationTable builtTue Apr 18 17:19:48 UTC 2017 by CanReg5

Data for the year 2015 was initially analysed using Canreg-5 to estimate the incidence and ASR for the various tumours. Data was also exported to Microsoft Excel for further analysis using Epi Info version 7.1.4. Microsoft Excel was used to generate charts and graphs. Aggregated data for the years 2013 and 2014 were also analysed for trends in cancer incidence and ASR.

## Results

### Incidence of cases of cancer

Overall, a total of 736 cancer cases were recorded among the residents of Kumasi for the year 2015. Based on the estimated population of 2,311,480 for the year 2015 for the city of Kumasi, the incidence among female residents was estimated at 54.1 per 100,000 (compared to 37.1 per 100,000 in males). Among males, the highest cancer incidence was for prostate cancer with an incidence of 10.5 per 100,000. Among females, breast and cervical cancers recorded the highest incidences of 16.1 per 100,000 and 13.7 per 100,000 respectively. The age specific incidence rates by sex for the various cancers are as shown in Tables 2 and 1.

**Table 2:** Frequency, age specific incidence rates, average annual crude incidence rates and ASR by site in males in Kumasi, 2015

Site /Age Group	All ages	Age Unknown	0–4	5–9	10–14	15–19	20–24	25–29	30–34	35–39	40–44	45–49	50–54	55–59	60–64	65–69	70–74	75+	Crude Rate	%	Cum (0–74)	ASR	ICD (10th)
Tongue	1	0	–	–	–	–	–	–	–	–	–	–	–	–	4.8	–	–	–	0.1	0.4	0.02	0.2	C01–02
Mouth	4	0	–	–	–	–	–	–	–	–	1.9	2.4	–	–	–	–	7.5	5.3	0.4	1.5	0.06	0.5	C03–06
Salivary glands	1	0	–	–	–	–	–	–	–	–	–	–	–	–	–	–	7.5	–	0.1	0.4	0.04	0.2	C07–08
Other oropharynx	3	0	–	–	–	–	–	–	–	–	–	–	–	4.1	4.8	–	7.5	–	0.3	1.1	0.08	0.5	C10
Nasopharynx	4	0	–	–	–	0.8	–	1.2	1.4	–	1.9	–	–	–	–	–	–	–	0.4	1.5	0.03	0.4	C11
Oesophagus	2	0	–	–	–	–	–	–	–	–	1.9	2.4	–	–	–	–	–	–	0.2	0.7	0.02	0.3	C15
Stomach	10	0	–	–	–	–	–	–	–	1.6	–	–	2.7	12.3	4.8	24.7	–	5.3	0.9	3.7	0.23	1.8	C16
Colon	3	0	–	–	–	–	–	–	–	1.6	–	–	2.7	–	4.8	8.2	–	–	0.3	1.1	0.07	0.5	C18
Rectum	6	0	–	–	–	–	–	–	2.7	1.6	1.9	–	2.7	4.1	–	–	–	–	0.5	2.2	0.07	0.7	C19–20
Anus	2	0	–	–	–	–	–	–	–	–	–	–	–	–	–	–	–	–	0.2	0.7	0.03	0.3	C21
Liver	58	0	0.6	–	–	–	–	1.2	6.9	17.8	15.1	14.3	27.4	32.9	4.8	32.9	7.5	–	5.2	21	0.82	7.6	C22
Gallbladder etc.	1	0	–	–	–	–	–	–	–	–	–	–	–	–	–	8.2	–	–	0.1	0.4	0.04	0.2	C23–24
Pancreas	7	0	–	–	–	–	–	–	–	–	1.9	–	–	–	4.8	8.2	–	21.3	0.6	2.6	0.07	1	C25
Nose, sinuses etc.	3	0	–	–	–	–	–	–	2.7	–	–	–	2.7	–	–	–	–	–	0.3	1.1	0.03	0.3	C30–31
Larynx	3	0	–	–	–	–	–	–	–	–	–	–	–	–	4.8	–	7.5	–	0.3	1.1	0.07	0.4	C32
Trachea, bronchus and lung	5	0	–	–	–	–	–	–	–	–	–	–	5.5	4.1	–	8.2	7.5	–	0.5	1.8	0.13	0.8	C33–34
Other thoracic organs	2	0	–	–	–	–	–	–	–	–	–	–	–	4.1	–	–	7.5	–	0.2	0.7	0.06	0.3	C37–38
Bone	3	0	–	–	0.7	–	–	1.2	–	–	–	2.4	–	–	–	–	–	–	0.3	1.1	0.02	0.3	C40–41
Other skin	6	0	–	–	–	–	1.0	–	1.4	1.6	1.9	–	–	–	–	–	–	–	0.5	2.2	0.07	0.7	C44
Kaposi sarcoma	2	0	–	–	–	–	–	–	–	–	–	2.4	2.7	–	–	–	–	–	0.2	0.7	0.03	0.3	C46
Connective and soft tissue	11	0	0.6	–	2.2	–	1.0	1.2	–	3.2	–	4.8	2.7	–	–	–	–	–	1	4.1	0.08	1.1	C47,C49
Breast	7	0	–	–	–	–	1.0	–	–	–	3.8	–	–	4.1	–	–	–	–	0.6	2.6	0.04	0.8	C50
Prostate	67	0	–	–	–	–	–	–	1.4	–	–	2.4	2.7	16.5	57.2	107.0	52.8	138.5	6	24	1.22	10.5	C61
Testis	1	0	–	–	–	–	–	–	–	–	1.9	–	–	–	–	–	–	–	0.1	0.4	0.01	0.1	C62
Kidney	1	0	–	–	–	–	–	–	–	–	–	–	–	–	–	8.2	–	–	0.1	0.4	0.04	0.2	C64
Bladder	5	0	–	–	–	–	–	–	1.4	–	1.9	–	–	–	–	–	15.1	5.3	0.5	1.8	0.09	0.6	C67
Eye	4	0	2.5	–	–	–	–	–	–	–	–	–	–	–	–	–	–	–	0.4	1.5	0.01	0.3	C69
Brain, nervous system	1	0	–	–	–	–	–	–	–	–	–	2.4	–	–	–	–	–	–	0.1	0.4	0.01	0.1	C70–72
Thyroid	1	0	–	–	–	–	–	–	–	–	–	–	–	–	–	–	7.5	–	0.1	0.4	0.04	0.2	C73
Adrenal gland	2	0	0.6	–	–	–	–	–	–	–	–	–	–	–	–	–	7.5	–	0.2	0.7	0.04	0.2	C74
Hodgkin disease	2	0	–	–	–	–	1.0	–	–	–	–	–	2.7	–	–	–	–	–	0.2	0.7	0.02	0.2	C81
Non-Hodgkin lymphoma	25	0	1.9	2.7	1.5	0.8	1.0	3.5	1.4	1.6	3.8	2.4	2.7	8.2	4.8		7.5	5.3	2.3	9.2	0.22	2.5	C82–85,C96
Lymphoid leukaemia	3	0	–	–	–	–	–	–	–	–	–	–	–	–	–	–	7.5	–	0.3	1.1	0.04	0.3	C91
Myeloid leukaemia	4	0	–	–	–	0.8	–	–	–	–	3.8	–	–	–	–	–	–	–	0.4	1.5	0.03	0.4	C92–94
Other and unspecified	17	0	–	–	0.7	–	3.0	1.2	–	3.2	5.7	9.5	2.7	12.3	4.8	–	–	–	1.7	7	0.22	2.3	O&U
All sites	277	0	7.5	2.7	5.9	2.5	7.9	9.3	19.2	34.0	47.2	47.7	60.4	111.1	100.1	205.8	150.9	197.1	25.1	100	4.09	37.1	ALL
All sites but C44	271	0	7.5	2.7	5.9	2.5	7.9	9.3	19.2	34.0	47.2	47.7	60.4	102.9	100.1	205.8	150.9	197.1	24.5	–	4.02	36.4	ALLbC44

Females accounted for 62.4% of all cases. The overall incidence of cancer in Kumasi for 2015 was 46.1 per 100,000. The mean age of all cases was 51.3 years (with a range of 1 to 99 years). The modal and median ages were 65 and 53 years respectively. The mean age among female cases was 51.8 years (with modal, median and range of 54 years, 53 years and 1–99 years respectively).

#### Top five cancers

Overall, breast cancer was the commonest cancer recorded. Breast cancer accounted for 19.7% of all cancer cases in Kumasi in 2015 followed by cervical and liver cancers accounting for 14.7 and 11.4% respectively (Table 3).

**Table 3:** Top 5 cancers in Kumasi for both males and females, 2015

Cancer	Frequency	%
Breast	145	19.7
Cervix uteri	108	14.7
Liver	84	11.4
Prostate gland	66	9.0
Ovary	30	4.1

Among females, the commonest cancer recorded was breast cancer (accounting for 30.0% of all cases) followed by cervical (23.4%), Ovarian (6.3%) and Liver (6.1%) cancers (Tables 4 and 5).

**Table 4:** Top 5 female cancers in Kumasi, 2015

Cancer	Frequency	%
Breast	137	30.0
Cervix uteri	107	23.4
Ovary	29	6.3
Liver	28	6.1
Non-Hodgkin Lymphoma	21	4.6

**Table 5:** Ranking of Cancer Cases in Kumasi for 2012 and 2015 by sex

Sex	Both Sexes		Male		Female	
Rank/Year	2012	2015	2012	2015	2012	2015
1st	Breast	Breast	Liver	Prostate	Breast	Breast
2nd	Cervix	Cervix	Prostate	Liver	Cervix	Cervix
3rd	Ovary	Liver	Non-Hodgkin Lymphoma	Non-Hodgkin Lymphoma	Ovary	Ovary
4th	Liver	Prostate	Stomach	Stomach	Corpus uteri	Liver
5th	Prostate	Ovary	Lungs	Colorectum	Non-Hodgkin Lymphoma	Non-Hodgkin Lymphoma

Among males, prostate cancer was the commonest malignancy diagnosed in 2015 in Kumasi with a total of 66 cases accounting for 23.8% of cases recorded (Table 6). Other common male cancers recorded were Liver (20.2%) and Stomach (3.6%).

**Table 6:** Top 5 male cancers in Kumasi, 2015

Cancer	Frequency	%
Prostate gland	66	23.8
Liver	56	20.2
Non-Hodgkin lymphoma	25	9.2
Stomach	10	3.6
Colorectum	9	3.3

#### Basis of diagnosis

The histology of primary tumour was the commonest basis of diagnosis for the cancers diagnosed in Kumasi in 2015 (Table 7). A considerable proportion of cases (41.8%) were however diagnosed clinically.

**Table 7:** Basis of Diagnosis for cancers in Kumasi, 2015

Basis of diagnosis	Frequency	Percentage
Histology of primary	341	46.3
Clinical only	308	41.8
Clinical Investigation	60	8.2
Cytology or Haematology	22	3.0
Death Certificate Only	3	0.4
Specific tumour markers	2	0.3
Total	736	100.0

#### Trends in cancer cases, 2012 to 2015

**Fig. 1:**
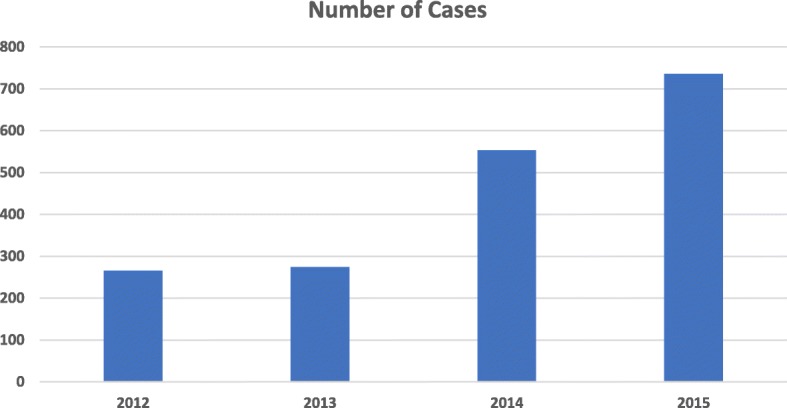
Cases of cancers reported in Kumasi, 2012 to 2015

The total numbers of cancers reported in Kumasi for the years 2012 to 2015 is shown in Fig. 1. The year 2015 reported the most cases followed by 2014 and 2013. The overall incidence of cancer in Kumasi for 2013 was 16.9 per 100,000 with an incidence of 23.2 per 100,000 among females and 10.9 per 100,000 in males. In 2014, the incidence was 35.4 and 30.4 per 100,000 among females and males respectively with the overall incidence estimated as 33.0 per 100,000 population.

## Discussion

At the inception of the Kumasi cancer registry, the operations of the registry were fraught with several challenges. Data capturing was difficult due in part to a scarcity of registrars as well as operational challenges during data collection. The attitude of health workers towards registrars collecting routine data was rather unwelcoming. However, progressive sensitisation of staff and the inclusion of other support staff in the collection of data has improved coverage and quality of data. This is demonstrated by the progressive increase in the number of cases reported. The increment (Fig. 1) may not necessarily imply an increase in cancer cases although globally the incidence of cancer is projected to increase [22]. There has been a decrease in the proportion of cases diagnosed histologically. This is partly due to improved data collection from sources such as birth and deaths registry and death certificates. The proportion of liver cancers captured has increased. A total of 736 cases were recorded for year 2015 out of which 85 were liver cancers representing 11.5%. In 2012, however, a total of 256 cases were recorded out of which 16 (6.3%) were liver cancers. Liver cancers are not usually biopsied hence diagnosis is often clinical and not based on histological examination.

Breast cancer remained the leading cause of cancer in Kumasi. This is consistent with our 2012 report on data from this registry [18] and the ranking of breast cancer as the leading cause of cancer-related morbidity in Ghana by the Global Burden of Disease Study (GBD) [23, 24]. The recently published findings from Globocan provide evidence that breast cancer is in fact the leading cause of cancer among females in Ghana. This is in contrast to an earlier publication in 2012 by Globocan in which cervical cancer was projected as the commonest cancer in Ghana [2, 25]. The GBD study draws on data from research and other published material and includes risk factor modelling to arrive at the estimates for the various cancers. Globocan, on the other hand, for countries where national or regional data are not available, makes estimates based on frequency data from the country as well as incidence data from neighbouring countries [26]. Consistent non-population based data on cancers in Ghana suggest breast cancer as the leading cause of cancer among females and cancer-related mortality in Ghana [8, 9]. Breast cancer being the leading cancer among females as found in this study is still inconsistent with evidence from the WHO which lists cervical cancer as the leading female cancer in Ghana [27]. Our results support the finding that the leading female cancer in Ghana is that of the breast and not of the cervix. The consistency of higher reporting recorded during the 4 years of operation of this PBCR provides sufficient evidence to support this, even though the data is from a single urban population. The lack of skilled healthcare workers to diagnose cervical cancer can contribute to poor reporting of cases. Gynaecologists and Pathologists, among other healthcare staff, are critical in diagnosing and managing cervical cancer and thus report cases. Pathologists in Ghana are unlikely to be selective in the tissue samples they report on; hence lack of skilled workforce for diagnosis cannot be responsible for any shortfall in cases of cervical cancer. Data from the Ghana College of Physicians and Surgeons suggests there are currently, in Ghana, more gynaecologists than there are general surgeons [28]. The relatively lower number of cervical cancer cases diagnosed is therefore unlikely to be due to a lack of expertise for diagnosing cervical cancer. If estimates from some studies are anything to go by, breast cancer can be assumed to be the leading cancer in Ghana [23–25].

The top five cancers in Kumasi have remained the same when 2012 and 2015 data were compared. There were however, some slight changes in terms of ranking [18]. The top five cancers recorded in Kumasi in 2015 were breast, cervical, liver, prostate and ovarian cancers, in descending order. Breast and cervical cancers were consistently ranked first and second in both years. Liver cancer was the third leading cancer in 2015 with ovarian cancer being the third leading cancer in 2012. The fourth and fifth ranked cancers in 2012 were liver and prostate cancers compared with prostate and ovarian cancers in that order in 2015. The top five cancers for both sexes in 2018 based on Globocan estimates were breast, cervix, liver, prostate and colorectum cancer [25]. There is some consistency between the top five cancers reported by Globocan based on estimates, and that reported based on local evidence from our population-based cancer registry.

We estimated the ASR for male cancers as 37.1 per 100,000 compared with 79.2 per 100,000 estimated in 2012 by Globocan [29]. This is quite significant in terms of variation between the estimated ASRs. An even higher difference in the ASRs among females was found when our data was compared with estimates from the Globocan. The overall ASR for females estimated for Ghana is 104.8 per 100,000 [29] compared with 54.1 per 100,000 recorded in our study. The recently published data from Globocan in 2018 estimate ASR for both sexes as 86.7 (compared to 46.1 in the present study) and 88.6 and 87.3 for males and females respectively [25]. It is important to note the differences in the ASRs estimated by Globocan over the two periods: while ASR among males reported by Globocan for Ghana in 2018 is higher than the figure from 2012 (88.6 per 100,000 vs 79.2 per 100,000), the reverse was the case among females. The ASRs estimated by this study are generally half of what has been estimated by Globocan for both sexes and are much higher than the rates we reported for 2012 [18]. The observed difference between the ASRs from our population-based registry and Globocan may be due to the fact that the cancer estimates from Globocan are based on mathematical modelling using population data from other countries and this mathematical modelling may not be entirely representative of the situation in Ghana. Comparing our ASRs to population-based cancer registries in neighbouring countries, they were lower when compared to rates in Abidjan, Ivory Coast where 83.7 per 100,000 was recorded among males while 98.6 per 100,000 was recorded for females over a three-year period, 1995–1997 [30]. Similarly, our rates were lower when compared to rates in Nigeria: 66.4 per 100,000 in men and 130.6 per 100,000 in women from the Ibadan PBCR and 58.3 per 100,000 in men and 138.6 per 100,000 in women from the Abuja PBCR over a 2 year period (2009–2010) [31]. Although our rates are lower than those from other West African countries, we like previous studies [30, 31], found that incidence rates are higher in females than males in West African populations. Although data quality (coverage and consistency) has improved, we do indicate that there may be some limitation with respect to the coverage of the registry. Our inability to include data from one private health facility within the city of Kumasi in the registry may account for the observed differences in the rates and may in addition be responsible for the low ASRs recorded in the present study. This should not impact the overall ranking of cancers in Kumasi as this private facility only provides care for patients with breast cancer. Another potential reason for the observed rate may be that some patients who are resident in Kumasi may be seen in health facilities outside Kumasi, most likely the capital city Accra where slightly better opportunities for cancer treatment (including private sector services) are available. Accra currently does not have a PBCR; hence such cases cannot be linked to their place of residence and subsequently included in our estimates. These challenges reinforce the need to continuously improve coverage of our registry while advocating for the establishment of more population-based cancer registries in Ghana to provide better evidence on the burden of cancers in general and aid planning for prevention and control efforts.

### Female cancers

In 2012, females accounted for 69.6% of all cancers recorded in Kumasi compared with 62.4% in 2015. The proportion recorded in 2012 in Kumasi is similar to the 70.2% recorded in Accra in 2012 [8]. Globally, the overall age standardized cancer incidence rate is almost 25% higher in men than in women, with rates of 205 and 165 per 100,000, respectively. In West Africa however, the incidence of cancer is reportedly higher among females [29]. This is consistent with the findings in our study where the majority of incident cases were female. Our findings are also similar to a study from Nigeria, another West African country, where females accounted for 66% of cancer cases from Abuja compared with 34% in males [31]. A sex ratio of 1:1 has been reported in some parts of Kenya [32].

### Liver cancer

Liver cancer was among the five commonest cancers reported in Kumasi in 2015. This is consistent with evidence from previous data from Kumasi [18]. Globally, liver cancer ranked sixth for cancer incidence and fourth for cancer deaths in 2015. In low socio-demographic index (SDI) countries, liver cancer has been ranked fourth for cancer incidence and first for cancer mortality. In middle and high middle SDI countries it ranked fourth and sixth, respectively, for cancer incidence but second for cancer mortality [24]. There is evidence of a high prevalence of hepatitis B infection among the adult population in Ghana [33]. Although there is a national vaccination programme on Hepatitis B for children, this was only implemented in 2002 [34] and has probably not yet had an impact in terms of an expected decline in liver cancer cases. The lowest age for liver cancer cases reported in this study for both sexes was within the 25–29 year age group. Per Ghana’s immunisation schedule [35], children who received the hepatitis B vaccine at the inception of the national programme will be aged 16 years or younger hence hepatitis B-related liver cancers may not as yet be affected by the introduction of the national vaccination programme. In any case the incidence of liver cancer among children age 16 years or younger in our PBCR was very low. We also note that in the 2012 data from Kumasi, liver cancer was the most common reported cancer among males in Kumasi. This may have been due to lower coverage of data from the registry at its inception in 2012. However, a consistent improvement in surveillance for cancers has been observed subsequently. Additionally, liver cancers are rarely confirmed by histological investigation in our setting. As a result, cases of non-primary liver cancers may have been diagnosed as primary liver cancers. These notwithstanding, liver cancer remains an important cancer in Ghana and requires active efforts to reduce its incidence.

### Prostate cancer

Prostate cancer was the commonest male cancer recorded in Kumasi in 2015. This is consistent with data from Globocan [36]. Prostate cancer is common among males of African descent and this is not a surprising finding although the incidence estimated in this study, 10.5 per 100,000 is lower than those from Globocan (13 per 100,000). There may be an underestimation of the cases of prostate cancer but it still highlights the consistency with respect to it being the leading cause of cancer among males in Ghana. Serum prostate specific antigen (PSA) has been reported to be sensitive for the diagnosis of prostate cancer in West Africans especially when combined with digital rectal examination (DRE) and transurethral ultrasound [37]. Further, Hsing et al. using a combination of PSA testing and DRE reported a high prevalence of screen detected prostate cancer among West Africans [38]. This calls for active efforts to screen eligible males for prostate cancer in Ghana.

### Cervical cancer

Cervical cancer is a common cancer among females in Kumasi. Although it has been estimated to be the leading cancer among females in Ghana [27, 36], there is consistent evidence from local data that breast cancer is the commonest cancer in Ghana [8, 18]. Risk factors for cervical cancer exist in Ghana. There is evidence of a high prevalence of HPV infection among sexually active women although the prevalence of the known oncogenic strains of HPV is not clearly known [5]. Establishing more surveillance systems on cancers can be one useful way of verifying this and so better inform cancer prevention and control activities in Ghana. There is also the need to institute a national HPV vaccination programme to reduce the incidence of cervical cancer among women in Ghana. A national screening programme will also be useful in reducing the incidence of advanced disease and improve clinical outcomes of cervical cancer cases in Ghana.

### Cancer cases by site

In 2012, data from the Kumasi Cancer Registry indicated that tumours overall were reported for 29 sites among males (compared with 34 sites in 2015) and this excludes 17 cases for which the sites were unspecified [18]. Similarly, among females, the numbers reported in 2012 were from 25 sites (compared with 36 sites in 2015) and this excludes 8 cases for which the sites were unspecified. These are indicative of a strengthening of the cancer surveillance system in Kumasi and not necessarily an increase in the number of cancer cases in general or specifically the emergence of tumours associated with sites not reported in 2012 in Kumasi. The diagnostic capabilities of Kumasi have also improved significantly since the inception of the registry in 2012. There are more pathologists providing services both within the government and private laboratories. This may also be contributory to the increased number of cancer sites reported in 2015.

### Limitations

Routine data collection by the Kumasi Cancer Registry does not include data from other possible sources of healthcare such as prayer camps, herbal medicine practitioners and other smaller or private health facilities. Although the proportion of cases, if any, seen at these sites may contribute to a higher incidence rate, we surmise that the probability of such cases occurring is very low. Operational challenges with obtaining data from one private facility which offers breast oncology services may also cause an underestimation of incidence of cancers in Kumasi.

## Conclusion

Reporting on cancer cases in Kumasi has seen significant improvement in data quality and coverage since its inception in 2012. Population-based cancer registries are feasible and there is the need for more of such registries to improve data on cancers in Ghana.

Our report indicates that breast cancer is the commonest female cancer in Ghana and is consistent with other evidence. The leading male cancer reported in this study is consistent with other local and international reports. Lung and skin cancers are rare in Kumasi.

## Abbreviations

AFCRN: African Cancer Registry Network
ASR: Age-standardised rates
CMV: Cytomegalovirus
EBV: Epstein Barr Virus
GBD: Global Burden of Disease Study
HBCR: Hospital-based cancer registries
HHV: Human Herpes Virus
HPV: Human Papilloma Virus
IARC: International Agency for Research on Cancer
KATH: Komfo Anokye Teaching Hospital
NCDs: Non-communicable diseases
PBCRs: Population-based cancer registries
SDI: Socio-demographic index
WHO: World Health Organisation
